# Disease Entities in Mucormycosis

**DOI:** 10.3390/jof5010023

**Published:** 2019-03-14

**Authors:** Alexandra Serris, François Danion, Fanny Lanternier

**Affiliations:** 1 Université Paris Descartes, Centre d’Infectiologie Necker Pasteur, IHU Imagine, Hôpital Necker-Enfants Malades, Assistance Publique-Hôpitaux de Paris (AP-HP), 75015 Paris, France; alexandra.serris@gmail.com (A.S.); francois.danion@aphp.fr (F.D.); 2 Centre National de Référence mycoses invasives et antifongiques, Unité de Mycologie Moléculaire, Institut Pasteur, 75015 Paris, France

**Keywords:** Mucormycosis, Mucorales, rhino-orbito-cerebral mucormycosis, pulmonary mucormycosis, health-care

## Abstract

Mucormycosis is an emerging life-threatening fungal infection caused by *Mucorales*. This infection occurs mainly in immunocompromised patients, especially with hematological malignancy, transplantation, or diabetes mellitus. Rhino-orbito-cerebral and pulmonary mucormycosis are the predominant forms. Interestingly, location is associated with the underlying disease as pulmonary mucormycosis is more frequent in hematological malignancy patients whereas rhino-orbito-cerebral mucormycosis is associated with diabetes. Cutaneous mucormycosis results from direct inoculation, mainly after trauma or surgery. Gastro-intestinal mucormycosis occurs after ingestion of contaminated food or with contaminated device and involves the stomach or colon. Disseminated disease is the most severe form and is associated with profound immunosuppression. Uncommon presentations with endocarditis, osteoarticluar or isolated cerebral infections are also described. Finally, health-care associated mucormycosis is a matter of concern in premature newborns and burn units. Clinical symptoms and CT scan findings are not specific, only the early reversed halo sign is associated with pulmonary mucormycosis. Circulating *Mucorales* DNA detection is a recent promising diagnostic tool that may lead to improving the diagnosis and prompting therapeutic initiation that should include antifungal treatment, correction of the underlying disease and surgery when feasible.

## 1. Introduction

Mucormycosis is an emerging invasive fungal infection with a high mortality rate caused by ubiquitous filamentous fungi belonging to the order of *Mucorales*. Reported incidence of mucormucosis has increased over the years, presumably because of an increase of the population at risk and improved diagnostic tools [1]. Mucormycosis occurs mostly in the context of an immunosuppression, such as hematologic malignancy, hematopoietic stem cell transplantation (HSCT), solid organ transplantation (SOT) and diabetes mellitus. The disease is characterized by an extensive necrotizing vasculitis, resulting in thrombosis and subsequent tissue infarction. Infection is the result of inhalation of sporangiospores, inoculation of wounds or ingestion. Subsequent angioinvasion by hyphae starts with a specific interaction with endothelial cells and can result in systemic dissemination of the disease [2].

Six distinct clinical syndromes can occur in susceptible hosts, which have been classified as rhino-orbito-cerebral, pulmonary, cutaneous, gastrointestinal, disseminated and uncommon presentations. The primary site of infection varies according to the different genera included in the order of *Mucorales* and to the underlying disease of the patients [3,4,5,6]. In a review of 929 reported cases of mucormycosis, pulmonary mucormycosis was the most frequent form of mucormycosis in patients with hematological malignancy and HSCT, followed by sinus infection [3]. In patients with SOT, pulmonary and sinus infections had a relatively similar frequency. Rhino-orbito-cerebral infections represented the majority of infections in patients with diabetes, whereas cutaneous mucormycosis constituted half the cases of mucormycosis in persons with no particular underlying condition. Isolated cerebral mucormycosis was the most common site of infection in intravenous drug users.

Prognosis remains poor with an overall mortality estimated between 32% and 70% in different studies [3,4,5,6,7,8]. Mucormycosis localization was associated with survival [4].

## 2. Rhino-Orbito-Cerebral Mucormycosis (ROCM)

**Figure 1:**
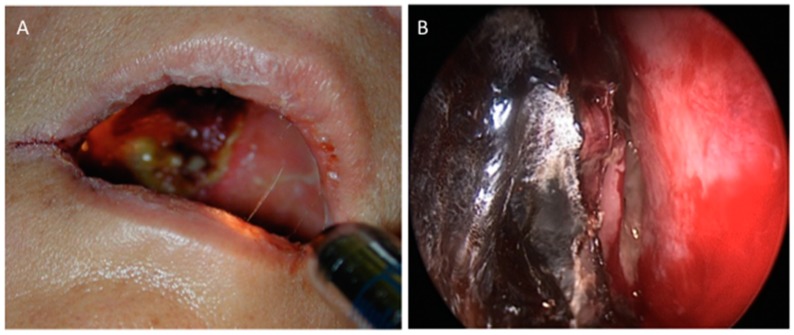
Necrosis in rhino-orbito-cerebral mycormycosis. (**A**): ROC Mucormycosis with palatal involvement and necrosis. (**B**): Endoscopic examination shows necrosis of the middle right meatus. Used with kind permission from B. Verillaud, personal data.

This is the most common clinical manifestation of mucormycosis. Infection begins with inhalation of spores that allows the fungus to spread to the paranasal sinuses. The infection can then rapidly extend to adjacent tissues: to the palate, the sphenoid sinuses, the orbits and the cavernous sinuses and eventually to the brain. Necrosis of these tissues, revealed by a black eschar, is a worrying sign of local extension (Figure 1).

In a meta-analysis of 175 ROCM cases published between 1994 and 2005, diabetes mellitus was the predominant underlying condition (64% of the cases), followed by hematological malignancy (15%) and renal diseases (13%) [9]. In two different studies, *Rhizopus* was predominantly associated with rhino-cerebral forms [4,5]. This finding might be explained by differences in virulence between genera in the order of *Mucorales*. In experimental studies, ketoacidosis has been found to predispose mice to *Rhizopus* spp. but not to *Lichtheimia spp.* infection [10]. This could be linked with the fact that the glucose-regulated protein 78 (GRP78), an endoplasmic reticulum chaperone protein of the HSP70 family, induced notably by elevated concentrations of glucose has been identified as the host receptor for *R. arrhizus* in endothelial cells in mice [2].

The main presenting symptoms were unilateral facial pain (86%), multiple cranial nerve palsies (68%), periorbital edema (59%), fever (50%) and diplopia (41%) in a study of 22 patients with ROCM [11]. Three clinical signs significantly affected survival and their presence should therefore prompt urgent treatment: chemosis, sixth cranial nerve palsy and cognitive disturbances.

Rapid differential diagnosis of ROCM and bacterial orbital cellulitis (BOC), much more frequent, is crucial for prevention of extension to orbital and cerebral tissues but can be challenging as early clinical signs are similar in both diseases. In a case-controlled study comparing 14 ROCM patients with 20 BOC patients, the authors found that extraocular muscle limitation was more frequent in ROCM patients, whereas eyelid swelling occurred more often in BOC patients [12]. Although a black eschar is often considered a characteristic finding of ROCM, it is probably a late sign as none of the patients with ROCM in this study had a black eschar upon their first nasal examination. 

**Figure 2:**
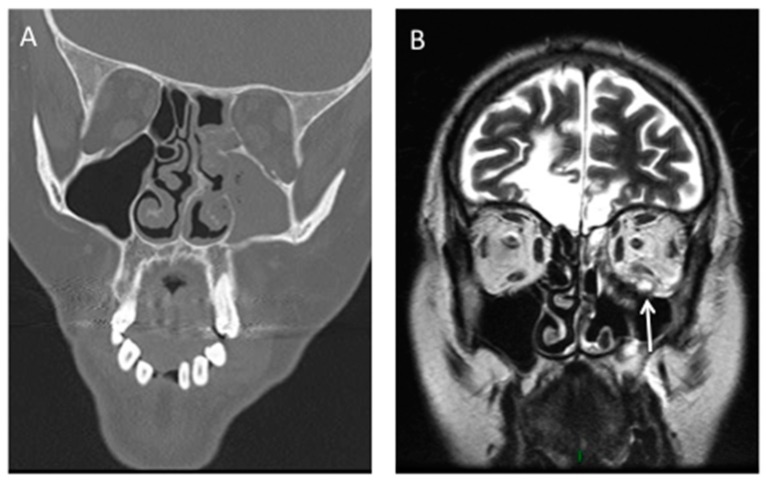
Rhino-Orbito-Cerebral mucormycosis in a diabetic patient. (**A**): CT scan shows left maxillary, ethmoidal and sphenoidal sinusitis complicated by osteolysis of the maxillary sinus. (**B**): Coronal T2-weighted MR image shows intra-orbital mass (arrow) compatible with fungal extension.

Abnormal paranasal sinus findings were detected on the CT scans of all 14 patients with ROCM (100%), whereas only 12 patients with BOC had sinus abnormalities (60.0%) (*p* = 0.011) [12]. Mucosal thickening and air-fluid level were found respectively in 13 (93%) and 1 (7%) patients with ROCM, whereas full opacification was never present. Bone destruction, extension to periorbital, intraorbital or intracranial tissues as well as vascular invasion should be particularly studied (Figure 2).

Optimal therapy of ROCM requires a multidisciplinary approach that relies on prompt institution of appropriate antifungal therapy, reversal of underlying predisposing conditions, and surgical debridement of devitalized tissue [13]. The analysis on 22 ROCM patients particularly emphasized the impact of local control of infection by repeated surgical procedures on survival (on day 90, 0% vs. 75% of patients had died respectively, with or without local control (*p* < 0.0001)) [11].

ROCM mortality rate varies according to the extension of the infection and the timeliness of management. In a meta-analysis published in December 2018, the overall mortality rate was 41.5%, which is not significantly better than the previous series published in 1994 by Yohai et al [9,14]. Early initiation of medical treatment was related to better survival outcomes (61% if commenced within first 12 days of presentation, compared to 33% if after 13 days). In the “RetroZygo” study, the overall 90-day mortality rate was 22% among patients with ROCM but significantly varied according to the extension of the infection: mortality was 56% in cases of cerebral involvement, 20% for sino-orbital forms and 0% for isolated sinusitis [4].

## 3. Pulmonary Mucormycosis (PM)

Pulmonary mucormycosis is particularly associated with hematologic malignancies [15]. According to three recent studies conducted in Europe, North America and France, hematologic malignancies are the most prevalent underlying diseases in patients with PM (51%, 70% and 79% of the patients, respectively) and conversely lungs are the main location of mucormycosis in patients with hematologic malignancies (34%, 43% and 44% of the patients, respectively) [4,6,8]. PM is also the dominant form of mucormycosis observed in solid organ transplant recipients [16,17]. Among patients with hematologic malignancies, key risk factors of mucormycosis have been identified. Those risk factors included neutropenia (80%) followed by corticosteroids (26%), hematopoietic stem cell transplant (24%), diabetes (18%), and graft versus-host disease (GVHD) (10%) [4].

Symptoms of PM are nonspecific and include fever, cough, dyspnea and chest pain [15]. Lesions typically involve the parenchyma but may extend into the chest wall, pulmonary artery, aorta, mediastinum, or pericardium. Hemoptysis can be caused by invasion of pulmonary arteries [18]. In a review of localized pulmonary mucormycosis, Lee et al. found that 97% of the cases had visible endobronchial diseases such as stenosis, erythema mucosa, obstruction of airway, mucoid secretions, polypoid mass and granulation tissue [19]. 

**Figure 3:**
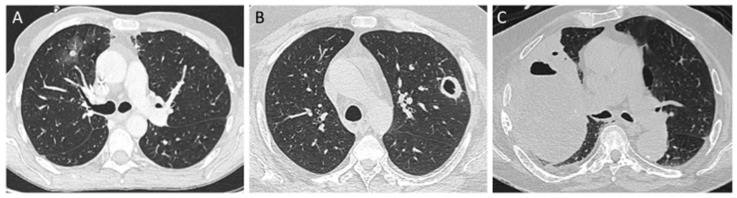
Different CT scan findings in pulmonary mucormycosis. (**A**): Axial pulmonary CT scan shows a nodule surrounded by ground-glass opacity in a patient with hematological malignancy. (**B**): Axial CT scan reveals excavated pulmonary nodule in a diabetic patient. (**C**): Axial CT scan shows voluminous mass with costal extension in a patient with lung cancer.

Symptoms consistent with PM in patients with risk factors should lead to prompt realization of a high-resolution computed tomography (CT). CT findings are not specific but may be suggestive of fungal infection with nodules (with or without halo sign or reversed halo sign), mass, cavitation, micronodules and pleural effusion (Figure 3). Notably, differential diagnosis between mucormycosis and invasive pulmonary aspergillosis (IPA) can be challenging as they share similar clinical and radiological presentations as well as host risk factors. An early pattern which seems to indicate the presence of mucormycosis is the reverse halo sign (RHS), a focal ground-glass opacity associated with a ring or crescent-shaped consolidation [20]. In a study comparing the CT scans of 24 patients with PM and of 96 patients with IPA, it was found to be more common in patients with PM (54%) than in those with aspergillosis (6%, *p* < 0.001), whereas some airway-invasive features, such as clusters of centrilobular nodules, peribronchial consolidations, and bronchial wall thickening, were more common in patients with aspergillosis [21]. However, in a recent study, where very early and serial thoracic CT scans were performed in neutropenic leukemic patients with PM, sequential morphologic changes could be observed: a reversed halo sign during the first 5 days of the disease followed by consolidation or nodule or mass with halo sign and, finally, central necrosis and air-crescent sign [22]. Moreover, RHS was identified in 78% of cases in neutropenic patients, whereas it was less frequent in non-neutropenic patients (31%). Reticulations inside ground-glass opacities were noticed in 87% of the cases with RHS. To differentiate PM and aspergillosis, it might also help to look for rhino-orbito-cerebral symptoms and to systematically perform a sinus scan, as sinus involvement is more frequent in mucormycosis than in aspergillosis [23]. 

As in other forms of mucormycosis, therapy relies on the combination of medical (antifungal drugs and control of the underlying disease) and surgical treatment when necessary [13]. Mortality of PM is higher than in other localized forms of mucormycosis (48% at day 90) [4]. In a recent review, *Cunninghamella spp.* was mostly isolated from PM and was associated with higher mortality than other Mucorales [5].

## 4. Cutaneous Mucormycosis

Cutaneous mucormycosis results from direct inoculation of fungal spores into damaged skin, which may lead to disseminated disease. The reverse (dissemination from internal organs to the skin) is very rare. In a review of 929 reported cases of mucormycosis, cutaneous involvement was the third site of mucormycosis infection in terms of frequency, involving 19% of the patients [3]. Most cases were localized to the skin, but deep extension to bone or muscle occurred in 24% of the cases and hematogenous dissemination from the skin to other noncontiguous organs developed in 20% of the cases. Hematogenous dissemination from other organs to the skin was noted in only 3% of the cases. A history of rupture of the skin barrier such as trauma (40%), surgery (15%), use of dressing (15%) or burns (6%) was reported in the majority of these patients. Cases of cutaneous mucormycosis are described after natural disasters (tornado, tsunami, volcanic eruption) and blast injuries during combat [24,25].

It is interesting to note that post traumatic mucormycosis represented the third cause of mucormycosis in the French “RetroZygo” study (18%) and the second cause (17%) in the European study conducted at the same time. [4,6]. It mostly affected immunocompetent patients (75% of the patients had no underlying disease in the literature review) [26]. Traffic accident (37%), domestic accident (15%) and natural disaster (13%) were the main forms of trauma. The clinical manifestations of cutaneous mucormycosis are diverse. The typical presentation of cutaneous mucormycosis is induration of the skin with surrounding erythema, rapidly progressing to necrosis (Figure 3). However, a nonspecific erythematous macule may also be the cutaneous manifestation of disseminated disease in an immunosuppressed patient [27,28]. The main symptoms were necrosis (76%), redness (48%), swelling (43%), purulent discharge (23%) and moldy appearance (22%). *Apophysomyces elegans, Lichthemia* and *Mucor species* were the main *Mucorales* isolates. *Mucorales* were often associated with bacterial infection (41% of the cases), which can delay the diagnosis of mucormycosis [26]. 

Skin or wound biopsies should be systematically performed as the diagnosis can be easily addressed by direct examination and culture.

Outcome is better than in other forms of mucormycosis (13–23% of mortality at day 90) [26]. Treatment is a combination of surgical procedures and systemic antifungal drugs. In RetroZygo study, surgery was performed in 94% of the cases with multiple debridements in 38% [26]. ECIL-6 recommends surgery for soft tissue disease (grade AII) in combination with amphotericin B [13].

## 5. Gastro-Intestinal Mucormycosis

Primary gastrointestinal disease is the least frequent form of mucormycosis. It can be acquired by ingestion of contaminated food such as fermented milk and dried bread products but also be healthcare-associated with contaminated devices. The stomach has been described as the most common site of infection, followed by colon, small intestine and esophagus [29]. In a retrospective series of 31 cases, the most common form was intestinal (52%) and then gastric disease (42%) [30]. The two main underlying conditions were SOT (52%) and hematologic malignancy (35%). Gastro-intestinal mucormycosis is also described in premature neonates [31]. Abdominal pain was the most frequent presenting symptom (68%) followed by gastrointestinal bleeding (48%) and change in bowel habits (10%) [30]. Fever was present only in 19%. A larger proportion of patients with gastric mucormycosis were SOT recipients (11/13), whereas those with intestinal mucormycosis were more likely to have a hematologic malignancy (12/16) (*p* = 0.002). Diagnosis can be suspected on endoscopic findings showing fungal mass or necrotic lesions that overlie an ulcerated area and that can lead to perforation and peritonitis. Optimal treatment is urgent surgery combined with intravenous amphotericin B. As symptoms of gastrointestinal mucormycosis are non-specific, diagnosis is often delayed or missed and mortality remains high at 57% [30].

## 6. Disseminated Mucormycosis

Disseminated infection is defined as infection involving at least two non-contiguous sites. In a prospective study conducted in 13 European countries between 2005 and 2007, 15% of patients presented with disseminated disease [6]. The most common sites of infection were lungs, sinus, soft tissues, central nervous system, liver and kidney. Patients with iron overload (especially those receiving deferoxamine), profound immunosuppression (such as recipients of HSCT having graft-versus-host-disease treated with corticosteroids or prolonged neutropenia) are the main groups at risk for disseminated mucormycosis. Systematic staging of the infection with cerebral MRI and sinus-thoraco-abdominal CT scan should be performed to diagnose disseminated mucormycosis. Compared with other clinical presentations, patients with disseminated disease had a highest mortality (58–79%) [4,6].

## 7. Uncommon Presentations

Other less common forms of mucormycosis include endocarditis, bone and joint infections, peritonitis and pyelonephritis. Notably, mucormycosis is a rare cause of endocarditis in intravenous drug users. Osteoarticular mucormycosis principally occurs after direct inoculation especially after trauma or surgical intervention [32]. Peritonitis has been described in patients undergoing continuous ambulatory peritoneal dialysis and isolated renal mucormycosis in intravenous drug users as well as renal transplant recipients [28].

Another rare manifestation of mucormycosis is isolated cerebral mucormycosis whereas typical central nervous system mucormycosis occurs after spread from paranasal sinus location or in disseminated diseases. In a review of 68 reported cases in the literature, most patients with isolated cerebral mucormycosis had a history of intravenous drug abuse (82%) [33]. Lesions were mostly localized in the basal ganglia (71.2%). The mortality rate was 65%.

## 8. Health-Care Associated Mucormycosis (HCM)

Health-care associated mucormycosis is a matter of concern, especially in neonatology, hematological, transplantation and intensive care units with burn patients. 

In a series of 169 cases of HCM identified between 1970 and 2008, major underlying diseases associated with HCM were solid organ transplantation (24%) (mostly because of graft-transmitted mucormycosis, involving 60% of SOT recipients), diabetes (22%), and severe prematurity (21%) [31]. Forty-one per cent of the cases were considered as surgical site infections (cardiovascular surgery being the most frequent procedure associated with HCM). Some other cases occurred in cluster and were linked to the use of contaminated devices (such as adhesive bandages, wooden tongue depressors, ostomy bags) or environmental reservoir (water circuitry and adjacent building construction) [31]. Recent outbreaks have been linked to contaminated hospital linens [34,35,36]. Skin was the most common localization (57%), followed by gastrointestinal tract (15%) in HCM [31]. *Rhizopus* was the most frequent genus identified (43%) as in other forms of mucormycosis. 

In intensive care units, burn patients appear to be particularly susceptible to fungal infection because of the loss of the skin barrier and acquired immune deficiency. In these patients, invasive wound mucormycosis is associated with an extremely poor prognosis despite a good tissue diffusion of amphotericin B. In a single-center study conducted between 2013 and 2016 in a specialized referral burn unit, the recorded mortality was 62% for patients with invasive wound mucormycosis [37].

## 9. Conclusions

Mucormycosis is an emerging life-threatening fungal infection characterized by host tissue infarction and necrosis that occurs mostly in immunocompromised patients. The distribution of risk factors varies across geographical regions, as hematological malignancy is the main risk factor in Europe and North America, followed by diabetes, trauma and SOT, whereas uncontrolled diabetes is the most common underlying disease found in studies in India or in Mexico [4,6,8,38,39]. Rhino-orbito-cerebral and pulmonary infections are the predominant forms. Its nonspecific clinical manifestations make differential diagnosis with other invasive fungal diseases difficult; the diagnosis is thus often delayed, which result in poor outcomes despite the availability of better diagnostic and therapeutic tools. Multidisciplinary approach is essential for prompt diagnosis and management of mucormycosis. 

## References

[1] Bitar D., Van Cauteren D., Lanternier F., Dannaoui E., Che D., Dromer F., Desenclos J.C., Lortholary O. (2009). Increasing incidence of zygomycosis (mucormycosis), France, 1997–2006. Emerg. Infect. Dis..

[2] Liu M., Spellberg B., Phan Q.T., Fu Y., Fu Y., Lee A.S., Edwards J.E., Filler S.G., Ibrahim A.S. (2010). The endothelial cell receptor GRP78 is required for mucormycosis pathogenesis in diabetic mice. J. Clin. Invest..

[3] Roden M.M., Zaoutis T.E., Buchanan W.L., Knudsen T.A., Sarkisova T.A., Schaufele R.L., Sein M., Sein T., Chiou C.C., Chu J.H. (2005). Epidemiology and outcome of zygomycosis: A review of 929 reported cases. Clin. Infect. Dis..

[4] Lanternier F., Dannaoui E., Morizot G., Elie C., Garcia-Hermoso D., Huerre M., Bitar D., Dromer F., Lortholary O. (2012). French Mycosis Study Group A global analysis of mucormycosis in France: The RetroZygo Study (2005–2007). Clin. Infect. Dis..

[5] Jeong W., Keighley C., Wolfe R., Lee W.L., Slavin M.A., Kong D.C.M., Chen S.C.A. (2019). The epidemiology and clinical manifestations of mucormycosis: A systematic review and meta-analysis of case reports. Clin. Microbiol. Infect..

[6] Skiada A., Pagano L., Groll A., Zimmerli S., Dupont B., Lagrou K., Lass-Florl C., Bouza E., Klimko N., Gaustad P. (2011). Zygomycosis in Europe: Analysis of 230 cases accrued by the registry of the European Confederation of Medical Mycology (ECMM) Working Group on Zygomycosis between 2005 and 2007. Clin. Microbiol. Infect..

[7] Pagano L., Valentini C.G., Posteraro B., Girmenia C., Ossi C., Pan A., Candoni A., Nosari A., Riva M., Cattaneo C. (2009). Zygomycosis in Italy: A survey of FIMUA-ECMM (Federazione Italiana di Micopatologia Umana ed Animale and European Confederation of Medical Mycology). J. Chemother. Florence Italy.

[8] Kontoyiannis D.P., Azie N., Franks B., Horn D.L. (2014). Prospective antifungal therapy (PATH) alliance(®): Focus on mucormycosis. Mycoses.

[9] Vaughan C., Bartolo A., Vallabh N., Leong S.C. (2018). A meta-analysis of survival factors in rhino-orbital-cerebral mucormycosis-has anything changed in the past 20 years?. Clin. Otolaryngol. Off. J. ENT-UK Off. J. Neth. Soc. Oto-Rhino-Laryngol. Cervico-Facial Surg..

[10] Ibrahim A.S., Spellberg B., Walsh T.J., Kontoyiannis D.P. (2012). Pathogenesis of mucormycosis. Clin. Infect. Dis..

[11] Vironneau P., Kania R., Morizot G., Elie C., Garcia-Hermoso D., Herman P., Lortholary O., Lanternier F. (2014). French Mycosis Study Group Local control of rhino-orbito-cerebral mucormycosis dramatically impacts survival. Clin. Microbiol. Infect..

[12] Son J.H., Lim H.B., Lee S.H., Yang J.W., Lee S.B. (2016). Early Differential Diagnosis of Rhino-Orbito-Cerebral Mucormycosis and Bacterial Orbital Cellulitis: Based on Computed Tomography Findings. PLoS ONE.

[13] Tissot F., Agrawal S., Pagano L., Petrikkos G., Groll A.H., Skiada A., Lass-Flörl C., Calandra T., Viscoli C., Herbrecht R. (2017). ECIL-6 guidelines for the treatment of invasive candidiasis, aspergillosis and mucormycosis in leukemia and hematopoietic stem cell transplant patients. Haematologica.

[14] Yohai R.A., Bullock J.D., Aziz A.A., Markert R.J. (1994). Survival factors in rhino-orbital-cerebral mucormycosis. Surv. Ophthalmol..

[15] Danion F., Aguilar C., Catherinot E., Alanio A., DeWolf S., Lortholary O., Lanternier F. (2015). Mucormycosis: New Developments into a Persistently Devastating Infection. Semin. Respir. Crit. Care Med..

[16] Sun H.Y., Aguado J.M., Bonatti H., Forrest G., Gupta K.L., Safdar N., John G.T., Pursell K.J., Muñoz P., Patel R. (2009). Pulmonary zygomycosis in solid organ transplant recipients in the current era. Am. J. Transplant..

[17] Park B.J., Pappas P.G., Wannemuehler K.A., Alexander B.D., Anaissie E.J., Andes D.R., Baddley J.W., Brown J.M., Brumble L.M., Freifeld A.G. (2011). Invasive non-Aspergillus mold infections in transplant recipients, United States, 2001–2006. Emerg. Infect. Dis..

[18] Pagano L., Offidani M., Fianchi L., Nosari A., Candoni A., Picardi M., Corvatta L., D’Antonio D., Girmenia C., Martino P. (2004). Mucormycosis in hematologic patients. Haematologica.

[19] Lee F.Y., Mossad S.B., Adal K.A. (1999). Pulmonary mucormycosis: The last 30 years. Arch. Intern. Med..

[20] Wahba H., Truong M.T., Lei X., Kontoyiannis D.P., Marom E.M. (2008). Reversed halo sign in invasive pulmonary fungal infections. Clin. Infect. Dis..

[21] Jung J., Kim M.Y., Lee H.J., Park Y.S., Lee S.-O., Choi S.-H., Kim Y.S., Woo J.H., Kim S.-H. (2015). Comparison of computed tomographic findings in pulmonary mucormycosis and invasive pulmonary aspergillosis. Clin. Microbiol. Infect..

[22] Legouge C., Caillot D., Chrétien M.L., Lafon I., Ferrant E., Audia S., Pagès P.B., Roques M., Estivalet L., Martin L. (2014). The reversed halo sign: Pathognomonic pattern of pulmonary mucormycosis in leukemic patients with neutropenia?. Clin. Infect. Dis..

[23] Bourcier J., Heudes P.-M., Morio F., Gastinne T., Chevallier P., Rialland-Battisti F., Garandeau C., Danner-Boucher I., Le Pape P., Frampas E. (2017). Prevalence of the reversed halo sign in neutropenic patients compared with non-neutropenic patients: Data from a single-centre study involving 27 patients with pulmonary mucormycosis (2003–2016). Mycoses.

[24] Neblett Fanfair R., Benedict K., Bos J., Bennett S.D., Lo Y.C., Adebanjo T., Etienne K., Deak E., Derado G., Shieh W.J. (2012). Necrotizing cutaneous mucormycosis after a tornado in Joplin, Missouri, in 2011. N. Engl. J. Med..

[25] Warkentien T., Rodriguez C., Lloyd B., Wells J., Weintrob A., Dunne J.R., Ganesan A., Li P., Bradley W., Gaskins L.J. (2012). Invasive mold infections following combat-related injuries. Clin. Infect. Dis..

[26] Lelievre L., Garcia-Hermoso D., Abdoul H., Hivelin M., Chouaki T., Toubas D., Mamez A.-C., Lantieri L., Lortholary O., Lanternier F. (2014). Posttraumatic mucormycosis: A nationwide study in France and review of the literature. Medicine (Baltimore).

[27] Skiada A., Petrikkos G. (2009). Cutaneous zygomycosis. Clin. Microbiol. Infect..

[28] Petrikkos G., Skiada A., Lortholary O., Roilides E., Walsh T.J., Kontoyiannis D.P. (2012). Epidemiology and clinical manifestations of mucormycosis. Clin. Infect. Dis..

[29] Agha F.P., Lee H.H., Boland C.R., Bradley S.F. (1985). Mucormycoma of the colon: Early diagnosis and successful management. AJR Am. J. Roentgenol..

[30] Dioverti M.V., Cawcutt K.A., Abidi M., Sohail M.R., Walker R.C., Osmon D.R. (2015). Gastrointestinal mucormycosis in immunocompromised hosts. Mycoses.

[31] Rammaert B., Lanternier F., Zahar J.-R., Dannaoui E., Bougnoux M.-E., Lecuit M., Lortholary O. (2012). Healthcare-associated mucormycosis. Clin. Infect. Dis..

[32] Taj-Aldeen S.J., Gamaletsou M.N., Rammaert B., Sipsas N.V., Zeller V., Roilides E., Kontoyiannis D.P., Henry M., Petraitis V., Moriyama B. (2017). Bone and joint infections caused by mucormycetes: A challenging osteoarticular mycosis of the twenty-first century. Med. Mycol..

[33] Kerezoudis P., Watts C.R., Bydon M., Dababneh A.S., Deyo C.N., Frye J.M., Kelley P.C., Kemp A.M., Palraj B.V., Pupillo G.T. (2018). Diagnosis and Treatment of Isolated Cerebral Mucormycosis: Patient-Level Data Meta-Analysis and Mayo Clinic Experience. World Neurosurg..

[34] Duffy J., Harris J., Gade L., Sehulster L., Newhouse E., O’Connell H., Noble-Wang J., Rao C., Balajee S.A., Chiller T. (2014). Mucormycosis outbreak associated with hospital linens. Pediatr. Infect. Dis. J..

[35] Cheng V.C.C., Chen J.H.K., Wong S.C.Y., Leung S.S.M., So S.Y.C., Lung D.C., Lee W.-M., Trendell-Smith N.J., Chan W.-M., Ng D. (2016). Hospital Outbreak of Pulmonary and Cutaneous Zygomycosis due to Contaminated Linen Items From Substandard Laundry. Clin. Infect. Dis..

[36] Sundermann A.J., Clancy C.J., Pasculle A.W., Liu G., Cumbie R.B., Driscoll E., Ayres A., Donahue L., Pergam S.A., Abbo L. (2018). How Clean Is the Linen at My Hospital? The Mucorales on Unclean Linen Discovery Study of Large United States Transplant and Cancer Centers. Clin. Infect. Dis..

[37] Legrand M., Gits-Muselli M., Boutin L., Garcia-Hermoso D., Maurel V., Soussi S., Benyamina M., Ferry A., Chaussard M., Hamane S. (2016). Detection of Circulating Mucorales DNA in Critically Ill Burn Patients: Preliminary Report of a Screening Strategy for Early Diagnosis and Treatment. Clin. Infect. Dis..

[38] Chakrabarti A., Chatterjee S.S., Das A., Panda N., Shivaprakash M.R., Kaur A., Varma S.C., Singhi S., Bhansali A., Sakhuja V. (2009). Invasive zygomycosis in India: Experience in a tertiary care hospital. Postgrad. Med. J..

[39] Corzo-León D.E., Chora-Hernández L.D., Rodríguez-Zulueta A.P., Walsh T.J. (2018). Diabetes mellitus as the major risk factor for mucormycosis in Mexico: Epidemiology, diagnosis, and outcomes of reported cases. Med. Mycol..

